
JOURNAL INFORMATION
==============================
NLM Title Abbreviation: ScientificWorldJournal
Journal ID: ScientificWorldJournal
NLM Journal ID: 101131163
Journal ID: TSWJ

The Scientific World Journal

ISSN: 2356-6140
EISSN: 1537-744X
Publisher: Wiley

ARTICLE INFORMATION
==============================
PMCID: PMC6009495
PMID: 29973780
DOI: 10.1100/tsw.2002.6
Article ID: 732196
Article version: 1
Subjects: Short Report

Mouse Genome Informatics: Mapping/Homology Data

Bradt D.
Bult C.J.
Blake J.A.
Kadin J.A.
Richardson J.E.
Eppig J.T.
The Jackson Laboratory, Bar Harbor, ME 04609, USA
Publication date: 2002
Electronic publication date: 2002 Jan 31
Volume: 2
First page: 11
Last page: 12
Copyright: Copyright © 2002 D. Bradt et al.
Copyright year: 2002
License: This is an open access article distributed under the Creative Commons Attribution License, which permits unrestricted use, distribution, and reproduction in any medium, provided the original work is properly cited.
License URL: https://creativecommons.org/licenses/by/3.0/

Funding: National Institutes of Health HG00330

==============================
Advances in Gene Technology: The Genome and Beyond –
Structural Biology for Medicine (Proceedings of the 2002 Miami
*Nature Biotechnology* Winter Symposium)
*TheScientificWorld* 2002, 2(S2), 11–12
ISSN 1532-2246; DOI 10.1100/tsw.2002.6

### MOUSE GENOME INFORMATICS: MAPPING/HOMOLOGY DATA

D. Bradt, C.J. Bult, J.A. Blake, J.A. Kadin, J.E. Richardson, J.T. Eppig, and the Mouse Genome Informatics Staff
The Jackson Laboratory, Bar Harbor, ME 04609
http://www.informatics.jax.org

**INTRODUCTION**. The Mouse Genome Informatics Web site at The Jackson Laboratory provides access to primary data and consensus representations of genetic, expression, phenotypic, and genomic information about the laboratory mouse[1,2,3]. Among many important aspects of this information are the mouse genome mapping data and mammalian comparative mapping data in The Mouse Genome Database (MGD).

**METHOD**. Mouse mapping data in MGD includes curated reports, primary research data from scientific publications, electronically submitted data, and data downloads from major genome centers. Genetic and physical mapping information, including summary haplotypes, are provided with recombination statistics and associated references. Genetic linkage maps derived from MGD consensus map positions, chromosome committee reports and various mapping panels can be selectively and flexibly generated and graphically displayed. Maps can be customized to contain selected subsets of mouse genetic markers and to display known homologs of any of the mammalian species for which MGD contains comparative mapping information[4].

MGD currently provides a scientifically curated data set of comparative mapping information for a select group of mammalian species, predominantly mouse, human, and rat. The availability of the whole genomic sequence for human and mouse (and soon, rat) has shifted the emphasis of data curation from literature-derived to curator-analyzed electronic-derived data. MGD has developed a set of criteria that support the assertions of homology to aid users in evaluating these annotations. In addition to providing homology data with supporting evidence and references, MGD provides graphical tools to view genome-wide comparative mapping data. Oxford grid displays graphically show chromosome-to-chromosome comparisons indicating the species-specific distribution of homologous genes. MGD also provides chromosomal comparative maps (for mouse vs. other mammals), as well as genome-wide comparative maps for mouse vs. human or rat.

**CONCLUSION**. Users have the ability to search and retrieve specific data types, sort search results, and print reports. MGD data are updated daily. MGD continues to evolve, expanding its data coverage, providing new data manipulation and display tools as well as promoting data integration with other scientific resources to meet the growing needs of the scientific community.

**ACKNOWLEDGMENT**. This work was supported by National Institutes of Health Grant HG00330.

### REFERENCES

1. Blake, J.A., Eppig, J.T., Richardson, J.E., Bult, C.J., Kadin, J.A., and the Mouse Genome Database Group (2001) Nucl. Acids Res. 29, 91–94.
2. Blake, J.A., Eppig, J.T., Richardson, J.E., Davisson, M.T., and the Mouse Genome Database Group (2000) Nucl. Acids Res. 28, 108–111.
3. Eppig, J.T., Blake, J.A., Davisson, M.T., Kadin, J.A., Richardson, J.E., and the Mouse Genome Database Group (2000) Lab Anim. 29, 39–43.
4. Davisson, M.T., Bradt, D.W., Merriam, J.J., Rockwood, S.F., and Eppig, J.T. (1999) The Mouse Gene Map. ILAR J. 39, 96–131.
